# Introgression among three rockfish species (*Sebastes* spp.) in the Salish Sea, northeast Pacific Ocean

**DOI:** 10.1371/journal.pone.0194068

**Published:** 2018-03-22

**Authors:** Piper L. Schwenke, Linda K. Park, Lorenz Hauser

**Affiliations:** 1 Conservation Biology Division, Northwest Fisheries Science Center, National Marine Fisheries Service, Seattle, Washington, United States of America; 2 School of Aquatic and Fishery Sciences, University of Washington, Seattle, Washington, United States of America; National Cheng Kung University, TAIWAN

## Abstract

Interspecific hybridization is often seen as a major conservation issue, potentially threatening endangered species and decreasing biodiversity. In natural populations, the conservation implications of hybridization depends on both on anthropogenic factors and the evolutionary processes maintaining the hybrid zone. However, the timeline and patterns of hybridization in the hybrid zone are often not known. Therefore, species conservation becomes a concern when recent anthropogenic changes influence hybridization and not if hybridization is part of a long-term process. Here, we use sequence data from one mitochondrial gene, three nuclear introns and one nuclear exon to estimate the direction, geographic extent, frequency and possible timeline of hybridization between three rockfish species (*Sebastes auriculatus*, *S*. *caurinus*, *S*. *maliger*) in the Salish Sea, Washington, USA. We show that (i) introgression occurred much more frequently in the Salish Sea than on the outer coast, (ii) introgression was highly asymmetrical from *S*. *maliger* into the other two species, (iii) almost 40% of individuals in the Salish Sea were hybrids, with frequency of hybrids increasing with isolation from the coast, and (iv) all hybrids were later generation backcrosses rather than F1 hybrids. Our results suggest long-standing low-level hybridization rather than recent onset of interbreeding because of human induced environmental change, possibly facilitated by specific environmental conditions in the sub-basins of the Salish Sea, and by differences in population sizes during recolonization of the area after the last glaciation. This rockfish hybrid system, with asymmetrical introgression and the maintenance of parental species, may prove useful to study both mechanisms that maintain species boundaries and that facilitate speciation in the presence of rapid environmental change.

## Introduction

Hybridization and introgression are often concerns for conservation of species impacted by fragmented or altered habitats [1, 2]; however, introgressive hybridization is also important for species evolution by providing a rich source of genetic variability [3–5]. This genetic variability provides opportunity for diversification and adaptability thus allowing colonization of new habitats and ecological niches [6]. Natural hybridization is especially common at the periphery of species ranges, where low population densities limit the availability of conspecifics as potential mates [7–11]. In these ecological peripheries or geographical range edges, hybrids theoretically encounter less competition with parental species, and introgressive hybridization can be maintained [6]. The evolutionary potential of hybrids and their parental species depend on specific mechanisms that influence the development and maintenance of hybrid zones in nature [12]. The conservation implications of hybridization in natural populations are therefore context dependent, depending both on anthropogenic factors and the evolutionary processes maintaining the hybrid zone [2].

Research on introgressive hybridization depends crucially on detection of hybrids and the accurate identification of later generation hybrids. Introgressive hybridization is often difficult to disentangle from other evolutionary signals in molecular data [13, 14]. Many closely related species share a portion of their genome either from retention of ancestral polymorphism or hybridization followed by introgression [10]. Hybrids are often detected during phylogenetic analysis when morphological species are not monophyletic [13] especially when geographic comparisons between hybrid zones and pure species ranges are possible [15]. Shared polymorphisms in areas of sympatry, together with reciprocal monophyly in areas of allopatry, provide clear evidence of hybridization [16, 17]. Additional evidence for long divergence times between species can strengthen the evidence for hybridization [15, 16, 18]; yet stochastic genetic processes in the genealogies of species necessitates systematic hypothesis testing to evaluate causes of paraphyly in gene trees [19, 20]. Hypotheses of hybridization can be tested using coalescence approaches with an isolation with migration (IM) model because the IM method accounts for these stochastic genetic variation by evaluating all locus genealogies consistent with the data [21, 22]. Interspecific gene flow among species can be quantified using the IM method and migration rates can then be compared between geographic regions to test for localized hybridization.

With the increasing power of molecular genetic approaches, hybridization has been documented in a growing number of marine species [23–27] including three closely related Pacific rockfish species, *Sebastes auriculatus*, *S*. *caurinus*, and *S*. *maliger* [28–30]. Like other species of *Sebastes*, these taxa are internal fertilizers and ovoviviparous, long lived and late maturing [31, 32]. The geographic distributions for these species mostly overlap on the Pacific Ocean coast from South California to the Gulf of Alaska with *S*. *auriculatus* more common than the other two species in Southern California and *S*. *maliger* more common than the other two species in Alaska [32]. The species distribution is mostly continuous, with one exception of *S*. *auriculatus* which has a break in coastal distribution between central Washington and North Vancouver Island, B.C. except for a population located in Puget Sound in the Southern Salish Sea [29, 32].

*Sebastes auriculatus*, *S*. *caurinus*, and *S*. *maliger* populations in the Salish Sea are isolated from the coast [31] and therefore experience distinct habitat and water quality compared with coastal populations. The Salish Sea is a semi-enclosed glacial fjord that formed towards the end of the Pleistocene epoch, approximately 12,000 years ago, and that consists of the Strait of Georgia (Canada), the Strait of Juan de Fuca and the Puget Sound Basin. Recruitment of some juvenile rockfish from outside the Puget Sound basin could be limited due to low surface water exchange from outside the main basin [33]. The Salish Sea is also home to genetically distinct populations of several marine species [31]. Indeed, *S*. *auriculatus*, *S*. *caurinus*, and *S*. *maliger* in the Salish Sea are highly differentiated from con-specifc outer coaster populations, suggesting little gene flow into or out of the Salish Sea.[28, 29, 31, 34] Adult *S*. *caurinus*, *S*. *maliger*, and *S*. *auriculatus* show strong site fidelity and remain sedentary in rocky reef areas. Although all three overlap in their geographic distribution, they tend to separate by depth, with *S*. *maliger* generally occupying deeper waters, *S*. *auriculatus* occupying shallow intertidal and estuarine habitats, and *S*. *caurinus* in intermediate depths [31, 32, 35].

The bathymetry, oceanography, and ecology of the Puget Sound may be particularly suited for the development of hybrid zones. Puget Sound consists of narrow, deep channels and shallow sills [36]. Compared to the coast and the San Juan Islands, Puget Sound has less reef habitat [37], lower salinity, more variable temperatures, and anoxic conditions; furthermore, this variability in water quality is most pronounced in Puget Sound sub-basins [38]. In recent decades, seasonal anoxia in some of the prime rockfish habitats in Puget Sound has become more severe, resulting in regular fish kills for example in Hood Canal [39]. These anoxic events, together with exploitation, derelict fishing gear and habitat change, have resulted in dramatic population reduction in some rockfish species within Puget Sound [40]. The rapid change of environmental conditions, together with a reduction in population sizes, may cause rapid changes in the dynamics of hybridization, which are potentially detectable with molecular markers.

While hybridization among these species of *Sebastes* has been described previously, the information on the extent of introgression across their range as well detailed data on direction of introgression is incomplete. Buonaccorsi et al. [34] found directional introgression from *S*. *maliger* into *S*. *auriculatus* in Puget Sound, while Seeb et al. [28] found evidence for introgression from *S*. *auriculatus* and *S*. *caurinus* into *S*. *maliger*, and Buonaccorsi in 2002 found no evidence of introgression in Puget Sound *S*. *caurinus* [29]. Here, we investigated spatial and temporal patterns of hybridization using multilocus sequence data and more expensive sampling to measure interspecific gene flow between *S*. *auriculatus*, *S*. *caurinus*, and *S*. *maliger* on the Pacific coast and the Salish Sea.

## Materials and methods

### Tissue collection and DNA preparation

**Fig 1:**
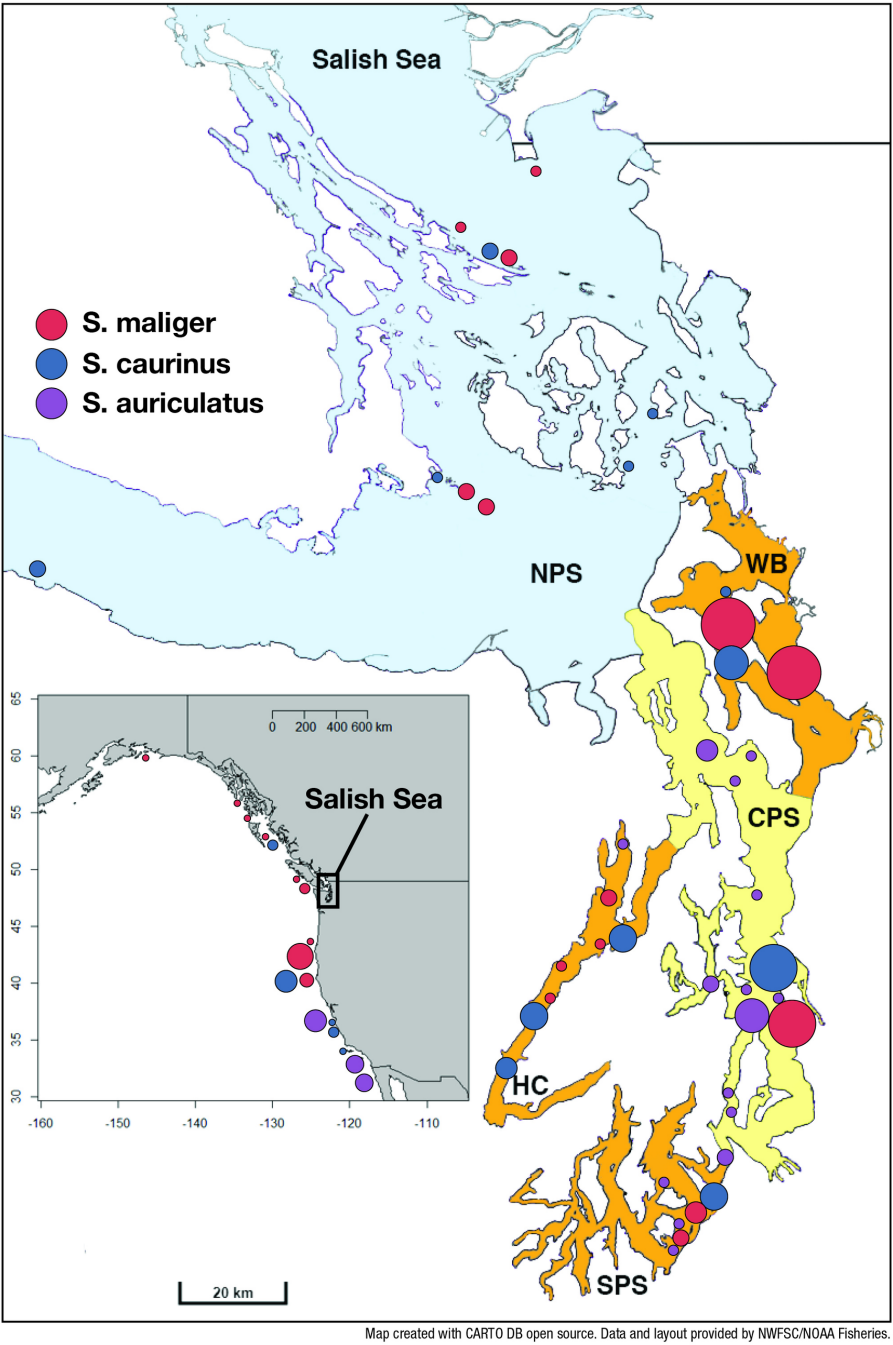
Fish collection localities and sampling regions. Localities for Salish Sea collection: Red circles are *S*. *maliger* (n = 40), purple circles are *S*. *auriculatus* (n = 24) and blue circles are *S*. *caurinus* (n = 33). Localities for coastal collections (inset): Red circles are *S*. *maliger* (n = 17), purple circles are *S*. *auriculatus* (n = 13), and blue circles are *S*. *caurinus* (n = 12). The size of the shape of the circles are proportional to the size of collection (1–8 individuals). More than one species collected from the same location is represented by adjacent circles. Major basins in the Salish Sea are represented by different colors and the lines at the mouth of each basin approximate locations of a natural, shallow sill. The color fill for each basin corresponds to the number of shallow sills that separate the basin from the outer coast. The sampling areas in the Salish Sea are South Puget Sound (SPS), Hood Canal (HC), Whidbey Basin (WB), and Central Puget Sound (CPS), and North Puget Sound (NPS). NPS includes the Strait of Georgia, San Juan Islands, and the Strait of Juan de Fuca.

Samples of *S*. *auriculatus* (n = 13), S. *caurinus* (n = 12), and *S*. *maliger* (n = 17) representing populations along the outer coastal range from Alaska to California were provided by the Southwest Fisheries Science Center (SWFSC) (Fig 1, Table 1). Tissue samples from the Salish Sea *S*. *auriculatus* (n = 24), S. *caurinus* (n = 33), and *S*. *maliger* (n = 40) were provided by the Washington Department of Fish and Wildlife (WDFW). The Salish Sea collection was mostly from the Puget Sound Basin but also included a few samples from the Strait of Juan de Fuca and the San Juan Archipelago (1997–2003, Fig 1, Table 1). Tissues were taken from whole specimens (University of Washington Fish Collection) of closely related species, *Sebastes dallii*, *S*. *atrovirens*, and *S*. *elongates*. All of these tissues samples are from specimens identified to species using morphologically distinguishing characteristics. In addition to known morphological species, tissues from three whole specimens suspected to be hybrids were also included for portions of the analyses (Table 2). The detailed locality data for all samples can be found in S1 Table.

**Table 1:** Tissue samples of *Sebastes auriculatus*, *S*. *caurinus*, *and S*. *maliger* from coastal and Salish Sea populations.

Species and Region	*S*. *auriculatus*		*S*. *caurinus*		*S*. *maliger*	
Coast Collection	N	Collection Year	N	Collection Year	N	Collection Year
Prince William Sound					1	1999
Southeast Alaska					2	1995
Queen Charlotte Island			2	1994	1	1994
West Vancouver Island					1	1998
Northern Oregon					3	1998
Southern Oregon					6	2002
Northern California			5	1999	3	1999
Central California	5	1999	3	1993		
Southern California	4	1999	1	1998		
Baja, Mexico	4	2000	1	2001		
**Total**	**13**		**12**		**17**	
**Salish Sea Collection**						
***S*. *auriculatus***						
North of Puget Sound			7	2002	8	2002
Central Puget Sound	17	2002	7	2002	6	2002
Whidbey Basin			5	1997, 2002	16	2002
Hood Canal	1	2002	10	2002	5	2002
South Puget Sound	6	2002	4	2001	5	2003, 2005
**Total**	**24**		**33**		**40**	

**Table 2:** Whole specimens of *Sebastes* species catalogued at the University of Washington Burke Museum Fish Collection (http://www.burkemuseum.org/ichthyology).

Sample ID	Latitude	Longitude	Collection year	Morphology	Region
UW 47319	47.727	-122.529	2004	*Sebastes caurinus* (suspected hybrid)	Central Puget Sound
UW 113205	47.145	-122.672	2005	*Sebastes caurinus* (suspected hybrid)	South Puget Sound
UW 113206	47.145	-122.672	2005	*Sebastes caurinus* (suspected hybrid)	South Puget Sound
UW114033	32.800	-117.275	2001	*Sebastes dallii*	Coastal (California)
UW 48830	48.175	-123.304	1999	*Sebastes elongatus*	Central Puget Sound
UW114048	34.414	-119.881	2000	*Sebastes atrovirens*	Coastal (California)

Genomic DNA was extracted from fin or muscle tissue using a Qiagen DNeasy 96 Tissue Kit on a Qiagen BioRobot 8000 (Hilden, Germany). Five regions of the genome were targeted using PCR amplification of the mitochondrial cytochrome b (*Cytb*), 5’ external transcribed spacer (*Ets*), *S7* ribosomal intron 2 (*S7*), malate dehydrogenase (*Mdh*), and malic enzyme (*Mep*). Primers for *Cytb*, *Ets*, and *S7* were obtained from the published literature (Table 3), primers for *Mdh* and *Mep* were originally designed using conserved regions *Mus musculus* and *Takifugu rubripes* genomes then redesigned for rockfish here. Conditions for PCR amplifications were specific to each locus, but generally PCR reactions were performed in a 40 μl reaction with 0.5 unit Go*Taq* DNA polymerase (Promega), 1X Go*Taq* Buffer, 200μM dNTP (Promega), each primer at 100–400 nM, and approximately 10–20 ng of genomic DNA. PCR was carried out for each locus with an initial denaturing step at 95°C for 2 minutes, followed by 32 cycles of 94°C for 40 seconds, locus specific annealing temperature and time (Table 3) and 60 second extension at 72°C.

**Table 3:** Locus data.

Locus	Primers	PCR Annealing Conditions	Reference	Locus Name	Genbank Accession Number
Cytb	F 5’ TGA CTT GAA RAA CCA YCG TTG 3’	58°C for 40 sec.	Rocha-Olivares et al. (1999)	Cytochrome b mitochondrial	JX886053-JX886194
	R 5’ATA TCA TTC TGG CTT AAT GTG 3’				
Ets	F 5’ CGG CCA TGG GCA GTT CAG G 3’				
	R 5’ATA TGC TTA AAT TCA GCG GG 3’				
S7	F 5’ AGCGCCAAAATAGTGAAGCC 3’	60 and 58°C for 60 sec.	Chow and Hazama (1998) and This paper	S7 Ribosomal, intron 2	JX886479-JX886620
	R 5’ GCCTTCAGGTCAGAGTTCAT 3'				
	C272 R 5’ CAT CTA CTG ACA CTT GTA TAC TA 3’ (internal with S7 – F)				
Mdh	F1 5’ CCT CTC TCA CTG CTG CTG AA 3’	61°C for 30 sec.	This paper	malate dehydrogenase, coding	JX886337-JX886478
	R1 5’ TTC TTC TCG ATG CCG TTC TT 3’				
	RF R2 5’ TCC CCA GAA GAA GAG GTG TG 3’ (internal with Mdh – F1)				
Mep	F 5’ GCT GTA ATG GAA TGG GCA TCC 3’	60°C for 60 sec.	This paper	malic enzyme, intron	JX886621-JX886762
	R 5’ AGC CTC TCC AGC TCC CTG G 3’				
	RF R2 5’ GGT TAA CTT TAT GGC ATT ATG AAG AA 3’ (internal with Mep – F)	60°C for 40 sec.			
	RF F2 5’ TTG GAA ACC ACA ATG CCT TC 3’				
	RF R3E 5’ CAC GGT AAA CAA TGA AGT AT CTG 3’	58°C for 60 sec			

### Sequencing

PCR products were purified using the Montage MultiScreen 96-well plate protocol (Millipore, Merck KGaA, Darmstadt, Germany) and sequenced in both directions using PCR primers and Big Dye Terminator Cycle Sequencing Ready Reaction version 3.1 (Thermo Fisher Scientific Inc. USA). Sequencing reactions were purified using CleanSeq Dye Terminator Removal Kit (Beckman Coulter Life Sciences, USA) and electrophoresed on an ABI3100 Genetic Analyzer (Thermo Fisher Scientific Inc. USA). The sequences were visualized, edited, and aligned using the software program Codon Code Aligner software version 3.7 (CodonCode Corporation, USA) which uses Phred Quality Scores [41]. Interpretations of heterozygous peaks were evaluated by eye from high quality sequence data (Q > 0.95). Locus alignments were evaluated for segregating sites in both the coastal and Salish Sea populations separately. All individuals used for analyses had complete sequence and locus data.

### Analysis

For phylogenetic analysis, *Sebastes elongatus* was used as the outgroup species and *S*. *dallii* and *S*. *atrovirens* as *Pteropodus* (Eigenman and Beeson 1893) ingroup control species. Both of these species are members of the sub-genus *Pteropodus* [30] and are closely related to *S*. *auriculatus*, *S*. *caurinus*, and *S*. *maliger*. The ingroup species were used to evaluate how well each gene tree resolved closely related species in the absence of hybridization. The best DNA mutation model was determined with ModelTest v3.7 [42] for each locus using the software’s default maximum likelihood settings. Individual sequences were collapsed into unique haplotypes and node labels were coded with unique haplotype identifier, species, population, haplotype count (see figure captions in S1 File). Phylogenies of the haplotypes were reconstructed using maximum likelihood heuristic search with random stepwise sequence addition implemented PAUP* 4.0 [43]. Introgression or ancestral polymorphisms were inferred from haplotypes found in genetic clades that did not correspond to their morphological identification.

The diploid nuclear data for each species group and locus were phased into haplotypes using the program PHASE implemented in DNAsp v5 [44] using the following MCMC parameters: burn-in of 10,000 steps, 10 step thinning intervals, and 100,000 iterations. The same software was used to calculate total haplotype number, number of segregating sites, number of unique haplotypes, haplotype diversity and its variance, nucleotide diversity, nucleotide divergence, population mutation rate per nucleotide site, and population mutation rate per gene[45]. We tested for evidence of intralocus recombination using the “4 gamete test” method [46] and selective neutrality at each locus using the Tajima’s D [47] in DNAspv5. In Arlequin v3.5 [48] we tested for population structure using *F*_*st*_ between species and populations from haplotype frequencies and performed an analysis of molecular variance (AMOVAs) among population and species.

To distinguish introgression from ancestral polymorphisms, an isolation with migration coalescence model [21] implemented in IMa2 [49] was used. IMa2 simultaneously estimates the marginal posterior probability densities (PPD) of mutation rate scaled population size, migration rate, and divergence time. The data partitions used for the IMa2 analysis were one or two of the largest non-recombining blocks from each nuclear locus sequence in addition to the entire *Cytb* locus sequence. In order to test for differences in hybridization levels between the three species in the Salish Sea compared to the coast, we analyzed the two data sets independently. The input parameters were identical in the two data sets: each locus was set for finite sites (HKY) mutation model and migration was only allowed between sampled populations. To get a rough estimate of population size (q) as a starting point for priors, we used the geometric mean of Watterson’s *θ* across loci to estimate the population mutation rate (per gene per generation) and took 5 x *θ* for q (IMa2.2 program documentation). The starting priors were initially set as follows: q = 10; m = 1; t = 4. Multiple short, independent MCMC runs were performed using different parameter priors to determine optimal prior settings that produced the most complete posterior probabilities density plots. The final MCMC input priors were uniform for each species and were set to q = 1, m = 4, and t = 1. The IMa2 MCMC chain was run for 100,000 burn-in steps with 1,000,000 run steps. Based on neutral expectation from maternal inheritance of a haploid mitochondrial genome compared to bi-parental inheritance of diploid nuclear loci, an inheritance scalar for the cytochrome b mitochondrial locus was set to 0.25 and the nuclear loci each were set to 1. The IMa2 data were evaluated to ensure that the MCMC parameter probability surface was well explored, the marginal distributions were adequately sampled, and that replicate MCMC run results were similar. Results for migration rate are reported as estimated effective number of migrants (2*NM*), which are independent of mutation rate (*μ*) [50]. In contrast, absolute estimates for population size and divergence time are dependent on mutation rate for each locus, and in the absence of mutation rate estimates could only be interpreted relatively between species and populations, assuming that mutation rates are similar among species.

Hybrids in coastal and Salish Sea populations of *S*. *auriculatus*, *S*. *caurinus*, and *S*. *maliger* were identified with *Structure* version 2.3.3 [51, 52]. We evaluated the program NewHybrids [53] to identify hybrids as well, but power was low due to the limited number of loci, and the hybrid class distinctions would not further objectives. Sequence data were formatted using xmfa2struct [54], which partitions each polymorphic site as a separate locus with the length of the sequence between polymorphic sites proportional to linkage distances. The linkage model in *Structure* was used to evaluate unphased, diploid data in 142 samples at 92 sites across 5 sequence fragments. The *Structure* analysis initiated a single MCMC chain with 10,000 burn-in steps, 100,000 run steps, 10 thinning interval steps, and 3 inferred clusters (k = 3). To determine the number of populations (k) that best fit the data, we evaluated the log likelihood score output for simulations using k = 1 to k = 7. The species for each genetic group was confirmed by morphological assignment in coastal samples in which hybridization is assumed to be rare. An individual fish was assumed to be a hybrid if its largest Q value was less than 0.90. This cut-off point was recommended in the literature [55]and was supported more recently by simulations [56]. The data from the three putative hybrid whole specimens were also included in the *Structure* analysis and grouped post-hoc with Salish Sea *S*. *caurinus* in order to validate our hybrid detection methods.

Spatial variation in hybrid abundance was evaluated by dividing Salish Sea collections based on naturally occurring boundaries at shallow sill inlets. The geographic regions were assigned as South Puget Sound (SPS), Central Puget Sound (CPS), Hood Canal (HC), Whidbey Basin (WB), and north of Puget Sound (NPS) including the Strait of Georgia, San Juan Islands, and the Strait of Juan de Fuca. The proportion of hybrids was compared between regions with a Fisher’s exact tests. Geographic regions were categorized by their isolation from the coast using the number of shallow sills and the Strait of Juan de Fuca as barriers for dispersal (or dispersal distance) to the coast (0 = coast; 1 = NPS; 2 = CPS; 3 = WB, HC, and SPS). The three sub-basins in Puget Sound were considered the same category. Correlations between location category and the proportions of hybrids were tested with Spearman's Rho.

## Results

DNA sequence data from five genes consisted of 293 to 785 nucleotides from 139 individuals across three species, each from the outer coast as well as the Salish Sea (Table 4). Each locus resolved 8 to 32 unique haplotypes, including the two ingroup and the outgroup species (GenBank: JX886053-JX886762). Haplotype diversity was greater in coastal than in Salish Sea populations in all species at *Cytb*, as well as in *S*. *maliger* at *S7*, and in *S*. *auriculatus* and *S*. *maliger* at *Mdh* (Table 4). However, Watterson *θ* (population mutation rate per gene) estimates for all three species were higher in the Salish Sea than the coast (S3 Table). The test for locus neutrality, Tajima’s D, was significant (p < 0.05) for *Cytb* sequences from Salish Sea *S*. *maliger* (Table 4), but not for the other populations or genes. No recombination was detected in the *Mdh* locus, but the other three loci contained varying numbers of non-recombining (NR) blocks across populations. Nucleotide size of each NR block were as follows: *S7* 181 and 145 bp, *Ets* 238 bp, and Mep 121 and 82 bp. The largest NR block from *Ets* and the two largest NR blocks from *S7* and *Mep* were used to create data partitions for the IMa2 analyses. These five NR blocks along with the complete sequence data from the *Mdh* and *Cytb* loci comprised the seven data partitions used in the IMa2.

**Table 4:** DNA polymorphism data for five genes across three species each with two populations.

Locus	nuc	species	pop	n	S	Nh	h + SD	*θ*	*Π*	K	TajimaD	NRB
*Cytb*	717	Sa	Co	13	6	7	0.879 ± 0.0057	0.0028	0.002	0.00435	-1.0217	n.a.
		Sa	SS	24	17	5	0.391±0.0157	0.0064	0.0035		-1.6503	n.a.
		Sc	Co	12	7	6	0.836±0.0079	0.0033	0.0027	0.00524	-0.7855	n.a.
		Sc	SS	33	13	7	0.81±0.0013	0.0045	0.0063		1.3329	n.a.
		Sm	Co	17	3	4	0.517±0.0175	0.0013	0.0009	0.00192	-0.8148	n.a.
		Sm	SS	40	22	8	0.484±0.0095	0.0076	0.0028		-2.1409*	n.a.
*S7*	542	Sa	Co	26	0	1	0	n.a.	0	0.00046	n.a.	n.a.
**		Sa	SS	48	6	4	0.125±0.0043	0.0025	0.0009		-1.6372	1
		Sc	Co	24	3	4	0.605±0.0062	0.0015	0.002	0.00305	0.8474	0
**		Sc	SS	66	7	8	0.742±0.0015	0.0027	0.0033		0.5456	2
		Sm	Co	34	8	12	0.877±0.0012	0.0036	0.0042	0.00357	0.4319	2
**		Sm	SS	80	6	9	0.761±0.0009	0.0022	0.0027		0.5048	2
*Ets*	293	Sa	Co	26	1	2	0.077±0.0049	0.0009	0.0003	0.00105	-1.1556	0
**		Sa	SS	48	1	2	0.422±0.0031	0.0008	0.0014		1.1852	0
		Sc	Co	24	2	3	0.518±0.0053	0.0018	0.0019	0.00244	0.0473	0
**		Sc	SS	66	5	6	0.53±0.0044	0.0036	0.0027		-0.5438	1
		Sm	Co	34	5	6	0.665±0.003	0.0042	0.0035	0.0041	-0.4433	1
**		Sm	SS	80	6	7	0.696±0.0014	0.0041	0.0046		0.2583	1
*Mdh*	730	Sa	Co	26	3	3	0.29±0.012	0.0011	0.0011	0.00079	-0.0453	0
**		Sa	SS	48	5	4	0.202±0.0059	0.0016	0.0005		-1.6408	0
		Sc	Co	24	1	2	0.237±0.011	0.0004	0.0003	0.00038	-0.2132	0
**		Sc	SS	66	2	3	0.246±0.0042	0.0006	0.0004		-0.5915	0
		Sm	Co	34	2	3	0.538±0.0057	0.0007	0.0008	0.00058	0.4271	0
**		Sm	SS	80	3	4	0.099±0.0021	0.0008	0.0002		-1.4907	0
*Mep*	785	Sa	Co	26	3	6	0.723±0.0053	0.001	0.0013	0.00209	0.7466	0
**		Sa	SS	48	7	12	0.768±0.003	0.002	0.0022		0.2544	3
		Sc	Co	24	3	4	0.636±0.0038	0.001	0.001	0.00174	-0.194	1
**		Sc	SS	66	7	7	0.788±0.0006	0.0019	0.0021		0.2912	1
		Sm	Co	34	9	9	0.839±0.0011	0.0028	0.0028	0.00301	-0.0594	1
**		Sm	SS	80	11	13	0.882±0.0002	0.0028	0.003		0.1204	3

nuc = number of nucleotides in analysis block; n = sample size; S = number of segregating sites; Nh = number of unique haplotypes; h + SD = Haplotype diversity and standard deviation (Nei 1987, equations 8.4 and 8.12 but replacing 2n by n); ***π*** = nucleotide diversity (Nei 1987, eq. 10.5); K = nucleotide divergence (Nei 1987, eq. 10.20), *θ* = pop mutation rate /site/generation (Nei 1987, equation 10.3); TajimaD = gene neutrality test (Table 2 in Tajima 1989)

* significant p < 0.05;

NRB = number of non-recombining blocks (Hudson and Kaplan 1985)

Species: Sa–*S*. *auriculatus*, Sc–*S*. *caurinus*, Sm–*S*. *maliger*. Populations (pop): Co–coast, and SS–Salish Sea

AMOVA results showed that most genetic variation could be explained by differences among species, with the exception of *Mep*, though differentiation between populations within species was significant for all loci (Table 5). *F*_*st*_ values between species in the Salish Sea were significant at all loci except *Mdh* between *S*. *auriculatus* and *S*. *maliger*; all species were significantly differentiated at all loci in the coastal populations (Table 6). Genetic differentiation between Salish Sea and the coastal population within each species were significant with the following exceptions: *S*. *auriculatus* at *S7* and *Mdh*, *S*. *caurinus* at *Cytb*, and *S*. *maliger* at *Cytb* and *Ets* (Table 6).

**Table 5:** AMOVA results (haplotype data).

**Source of Variation (%)**	**Cytb**	***S7***	***Ets***	***Mdh***	***Mep***
Among species	72.32	55.28	71.28	57.81	27.12
Among populations within species	4.69	3.20	1.08	4.43	6.33
Within populations	22.98	41.52	27.64	37.76	66.54
Fixation indices					
F_ct_ (species/total)	0.723	0.553	0.713	0.578	0.271
F_sc_ (population/species)	0.170**	0.072**	0.38*	0.105**	0.087**
F_st_ (population/total)	0.770**	0.585**	0.713**	0.622**	0.335**

*P<0.05,

**P<0.005

**Table 6:** *F*_ST_ values between species and populations.

Pop1	Pop2	Cytb	*S7*	*Ets*	*Mdh*	*Mep*	Average *F*_st_
Between populations within species							
SaCo	SaSS	**0.397**	0.028	**0.189**	0.025	**0.164**	**0.250**
ScCo	ScSS	0.141	**0.138**	**0.056**	**0.100**	**0.129**	**0.106**
SmCo	SmSS	0.032	**0.041**	0.017	**0.131**	**0.047**	0.073
Between species on coast							
SaCo	ScCo	**0.906**	**0.785**	**0.928**	**0.682**	**0.643**	**0.789**
SaCo	SmCo	**0.907**	**0.713**	**0.761**	**0.150**	**0.523**	**0.611**
ScCo	SmCo	**0.846**	**0.519**	**0.694**	**0.668**	**0.207**	**0.587**
Between species in Salish Sea							
SaSS	ScSS	**0.781**	**0.482**	**0.839**	**0.690**	**0.439**	**0.646**
SaSS	SmSS	**0.802**	**0.735**	**0.676**	0.005	**0.353**	**0.641**
ScSS	SmSS	**0.495**	**0.448**	**0.580**	**0.791**	**0.076**	**0.478**

Sa–*S*. *auriculatus*, Sc–*S*. *caurinus*, Sm–*S*. *maliger*, Co–coast, and SS–Salish Sea

All comparisons that are significant (p<0.05) are in bold; *F*_st_ calculation from Hudson et al. 1992b, equation 3

### Phylogenetic analysis

The DNA models of evolution were unique to each gene (Figure captions in S1 File). Each gene tree was evaluated for distinct clades and genetic clusters comprising haplotypes from morphological species given their geographic location. We identified haplotypes that were shared among species and “discordant” haplotypes in order to evaluate evidence of genetic introgression. A “discordant” haplotype was a haplotype found in a genetic clade or cluster that did not correspond to its morphological identification. Mostly, these haplotypes were shared among morphological species, although a few were unique haplotypes. The gene trees provided supportive phylogenetic information and showed signatures of hybridization but we could not directly identify hybrid individuals with confidence (See S1 File for more details).

### Coalescent analysis

**Fig 2:**
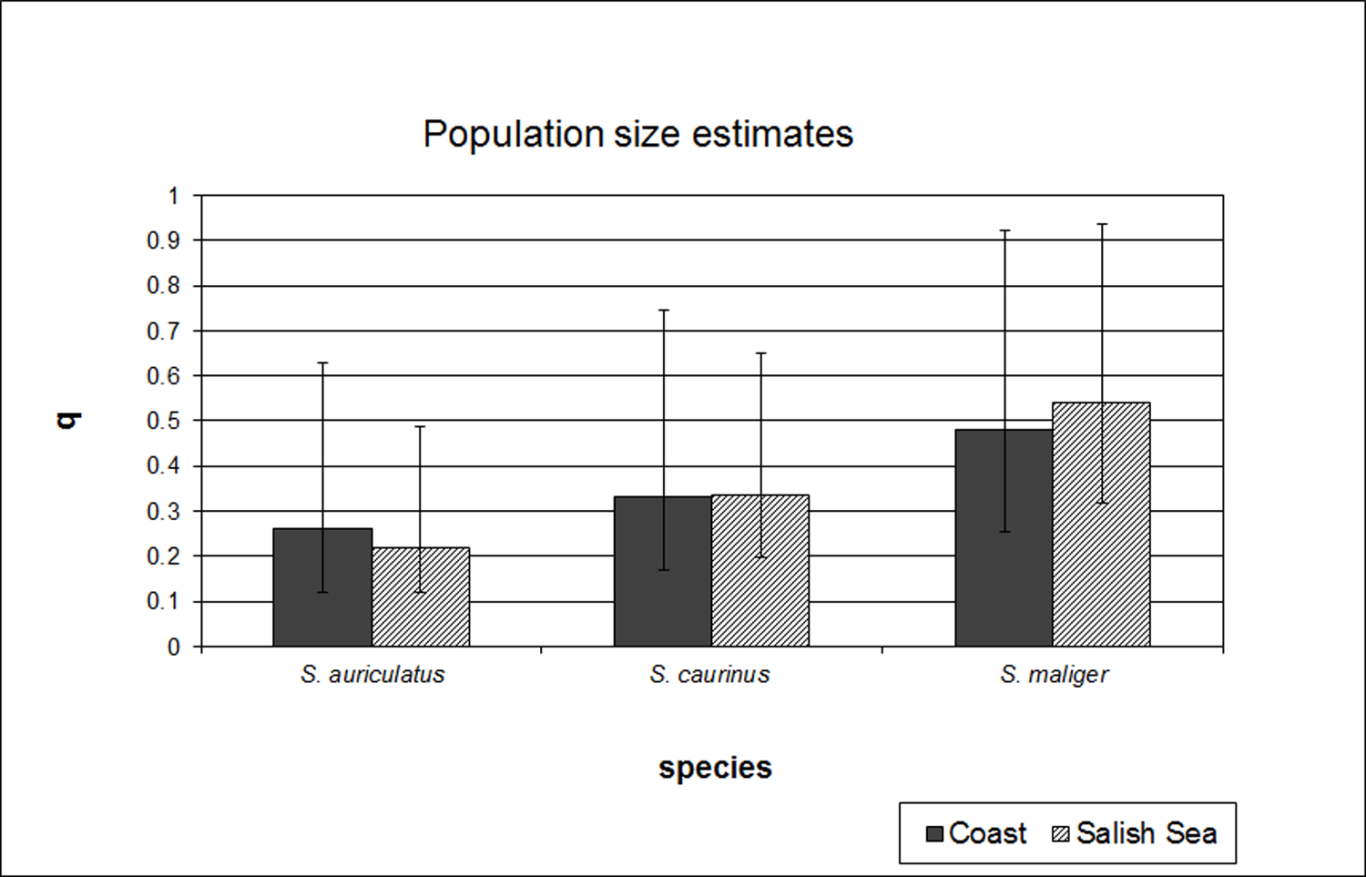
Population size parameter estimates (q) for *S*. *auriculatus*, *S*. *caurinus*, and *S*. *maliger* and between coastal and Salish Sea populations. The 95% CI are indicated with bars.

**Fig 3:**
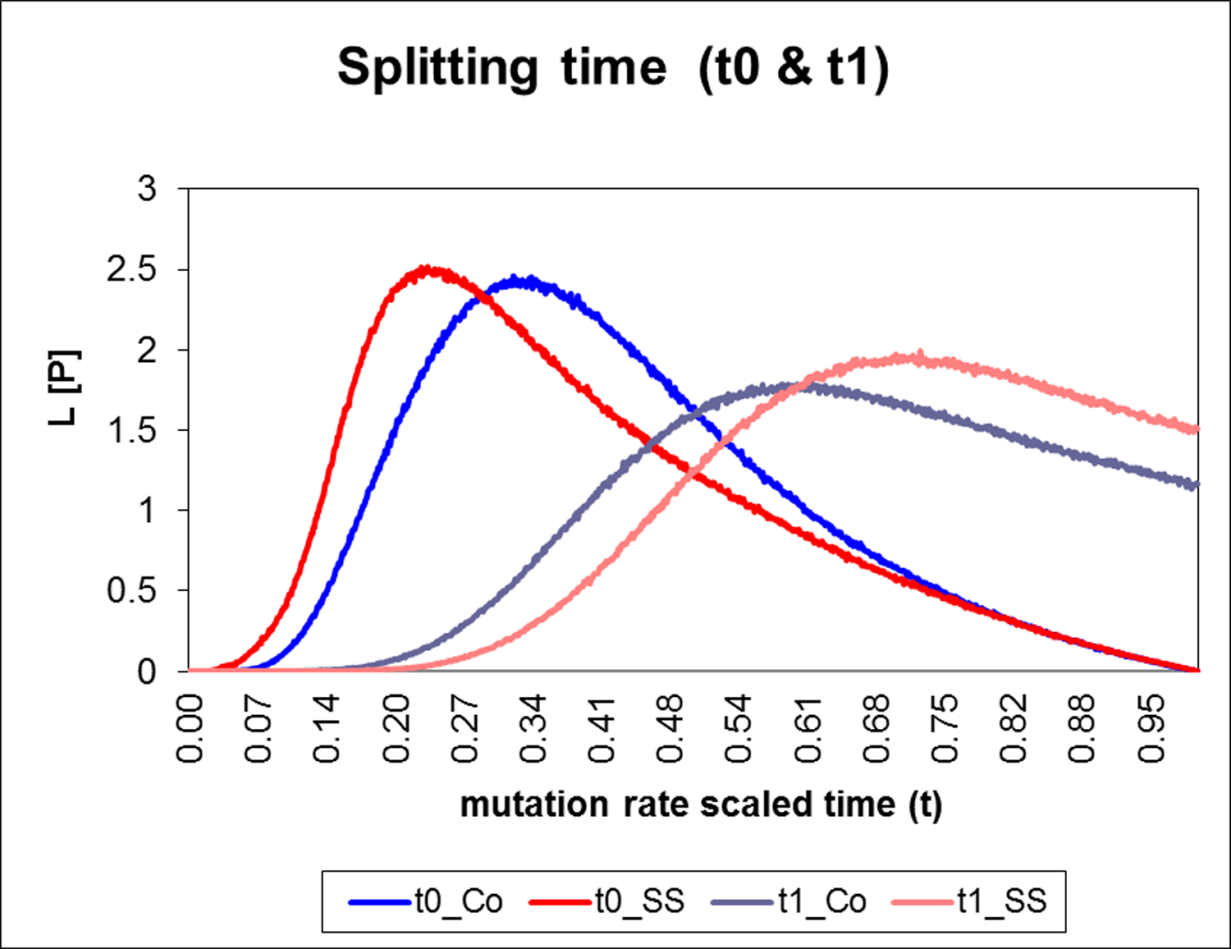
Posterior probability distribution (PPD) for mutation rate scaled splitting time in generations (t) between coastal (Co) and Salish Sea (SS). The t0 value is the spitting time for *S*. *caurinus* and *S*. *maliger* and t1 splitting time for *S*. *auriculatus* split from *S*. *caurinus* and *S*. *maliger*.

**Fig 4:**
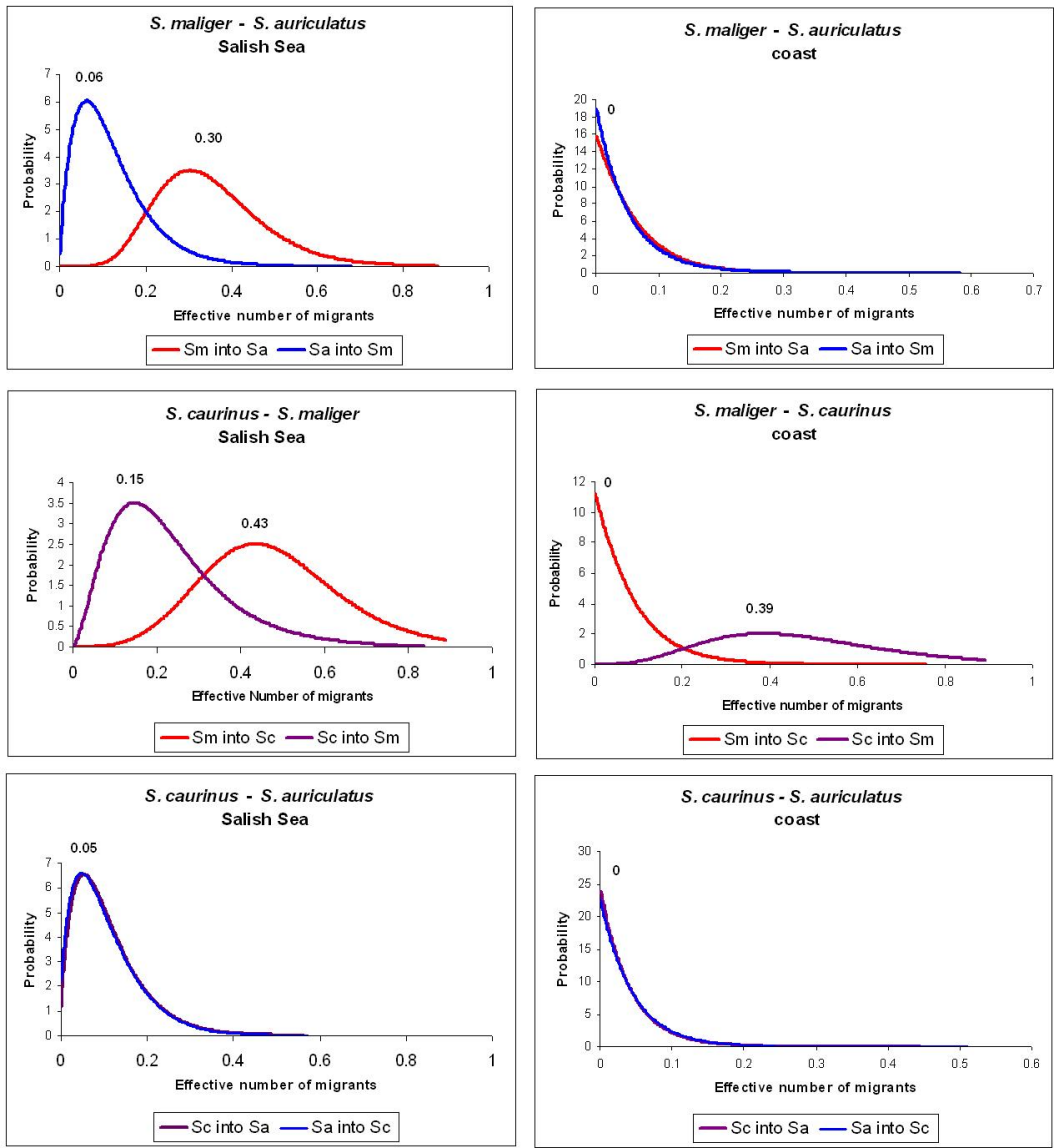
Posterior probability distribution (PPD) for estimated effective number of migrants (2NM) between species for the coastal and Salish Sea populations. Species are coded by two letters: Sa–*S*. *auriculatus*, Sc–*S*. *caurinus*, Sm–*S*. *maliger*. The PPD highest peak values are posted above the distribution.

The coalescent analysis indicated similar population sizes and interspecific divergence times for Salish Sea and coastal populations, but showed migration between species (i.e. introgression) only in the Salish Sea. Mutation rate-scaled population size parameters (q) varied between species (albeit not significantly) but not between Salish Sea and coastal populations within each species (Fig 2). Similarly, estimates of divergence times between *S*. *maliger* and *S*. *caurinus* (t0), and between *S*. *auriculatus* and the other two species were similar in the Salish Sea and the coast (Fig 3). In contrast, patterns of interspecific gene flow (hybridization) differed considerably between the two areas, with hybridization greater in the Salish Sea compared to the coast (Fig 4). The highest estimated effective number of migrants per generation (2NM) in the Salish Sea was from *S*. *maliger* into the other two species, while there was less hybridization between *S*. *caurinus* and *S*. *auriculatus*. Most of the migration rate estimates for the coast were close to zero except for *S*. *caurinus* into *S*. *maliger* (Fig 4).

### Admixture analysis

**Fig 5:**
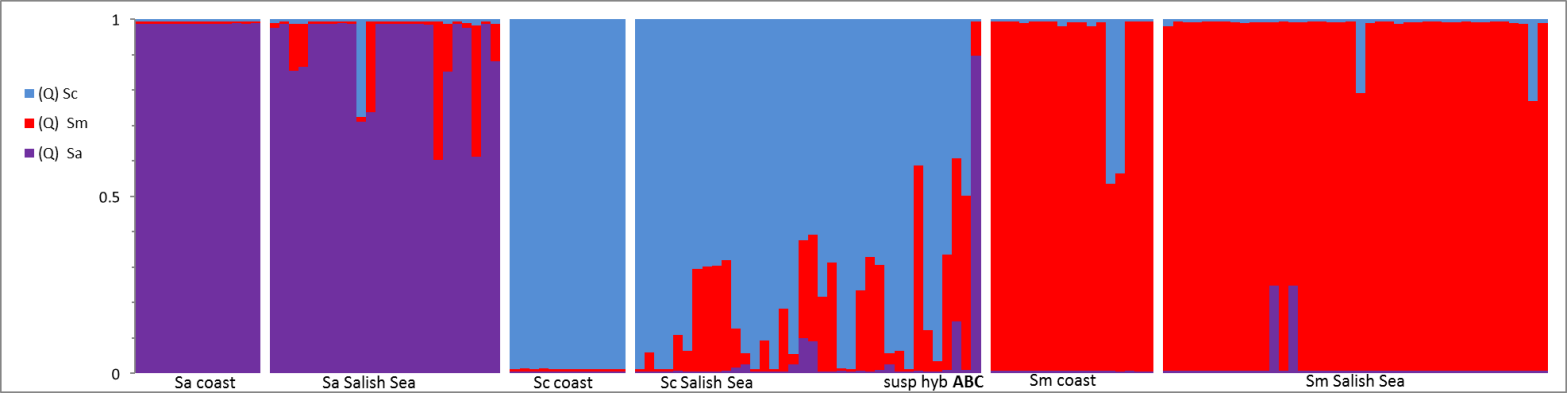
Ancestry coefficient (Q) from *structure* analysis for each individual to one of three genetic groups (k = 3). Each vertical bar represents a single individual and the colors shows the proportion of ancestry to each genetic group. The three genetic clusters are represented by purple as *S*. *auriculatus*, blue as *S*. *caurinus* and red as *S*. *maliger*. The results for each individual are arranged vertically by morphological species and population: 1 = *S*. *auriculatus* (coast), 2 = *S*. *auriculatus* (Salish Sea), 3 = *S*. *caurinus* (coast), 4 = *S*. *caurinus* (Salish Sea), 5 = *S*. *maliger* (Salish Sea), 6 = *S*. *maliger* (Salish Sea); A and B are *S*. *caurinus* hybrid whole specimens from SPS, and C is the *S*. *caurinus* putative hybrid whole specimen from CPS. The samples are approximately sorted from left to right on the figure by geography north to south.

The admixture results from the *Structure* analysis confirmed the mixed ancestry of the three putative hybrid whole specimens (Fig 5, S4 Table) and also verified morphological species identification, because they mostly assigned to their morphological species. The exception was a whole specimen morphologically identified as *S*. *caurinus* collected in CPS that appeared to be mostly pure *S*. *auriculatus* (UW 447319 in S4 Table). This curious result was verified with additional morphological examinations and replicate tissue sampling and sequence identification. Nevertheless, in general, morphological and genetic species identifications conformed to each other within this study.

The *Structure* analysis also showed that 29 out of 97 individuals (29.90%) caught in the Salish Sea were of mixed ancestry (Q<0.90). The relative proportions of hybrids were considerably higher in *S*. *caurinus* and *S*. *auriculatus* than in *S*. *maliger* (Fig 5, S4 Table). No hybrids were detected in coastal *S*. *caurinus* and *S*. *auriculatus*, and there were only two coastal *S*. *maliger* with evidence for hybridization (Fig 5, S4 Table). All hybrids appeared to be later generation backcrosses and none were F1 hybrids (Fig 5, S4 Table). Two *S*. *caurinus* from the tissue collection and one putative hybrid whole specimen had ancestry from all three species (Fig 5, S4 Table). Interestingly, the only evidence of *S*. *auriculatus* hybrid ancestry in *S*. *caurinus* was found in a single individual with three species ancestry (Fig 5). Two of the putative hybrids (whole specimens) collected in SPS appeared to have high levels of mixed ancestry (S4 Table, UW 113205 & 113206), and one tissue sample of *S*. *caurinus* (morphological specimen unavailable) from SPS appears to also have high levels of mixed ancestry (S4 Table, ScSS045).

### Spatial variation

**Fig 6:**
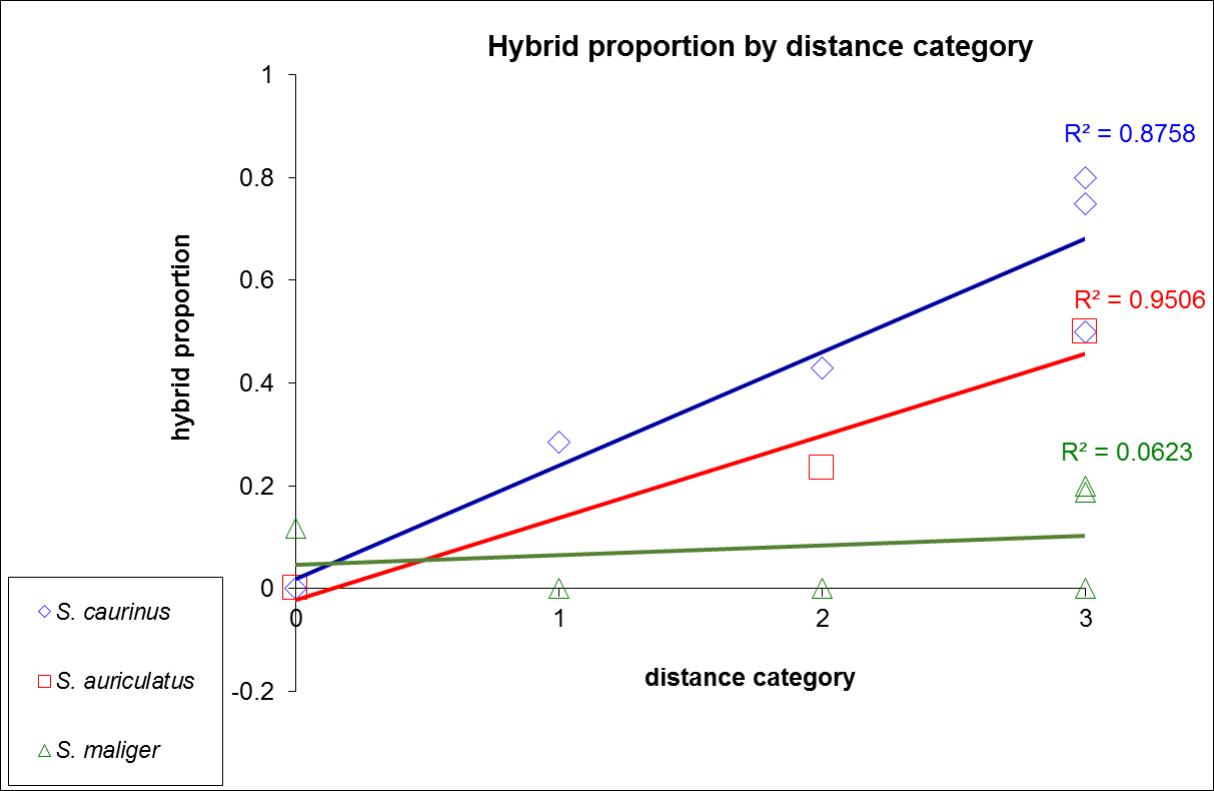
Proportion of hybrids in each species by sampling location. The proportion of hybrids depending on distance from the coast is measured by the number of sills. (0 = coast; 1 = North Puget Sound; 2 = Central Puget Sound; 3 = Whidbey Basin, Hood Canal and South Puget Sound). Spearman’s rank correlations are provided for each species.

A strong relationship was found between hybrid frequency and collection location. For all species combined, the proportion of hybrids (Table 7) in each geographic region within Puget Sound proper (south of Admiralty Inlet only) were significantly higher compared to the coast (Fisher’s exact test, p<0.05, S5 Table). However, the proportion of hybrids did not differ significantly among basins of Puget Sound (Fisher’s exact test p>0.10, S6 Table). Nevertheless, the proportion of hybrids in *S*. *caurinus* and *S*. *auriculatus* (but not in *S*. *maliger*) was higher in Puget Sound basins that were more isolated from the coast (i.e., the number of sills as barriers from the coast). The Spearman’s Rho statistic showed a strong positive relationship for *S*. *caurinus* and *S*. *auriculatus* (R^2^ = 0.88 and R^2^ = 0.95, Fig 6).

**Table 7:** Hybrid proportions by region and species.

Species and Region	*S*. *auriculatus*			*S*. *caurinus*			*S*. *maliger*		
	Hybrid	Non-Hybrid	Proportion hybrids	Hybrid	Non-Hybrid	Proportion hybrids	Hybrid	Non-Hybrid	Proportion hybrids
**Coast**	**0**	**13**	**0.00**	**0**	**12**	**0.00**	**2**	**17**	**0.12**
North of Puget Sound				2	7	0.29	0	8	0.00
Central Puget Sound	4	17	0.24	3	7	0.43	0	6	0.00
Whidbey Basin				4	5	0.80	3	16	0.19
Hood Canal	1	0	1.00	5	10	0.50	0	5	0.00
South Puget Sound	3	6	0.50	1	3	0.75	1	5	0.20
**Salish Sea Total**	**8**	**24**	**0.33**	**17**	**33**	**0.52**	**4**	**40**	**0.10**

North of Puget Sound includes the Strait of Georgia, San Juan Islands, and the Strait of Juan de Fuca.

## Discussion

Our results provided clear evidence for (i) asymmetrical introgression among three species of rockfishes in the Salish Sea, but not on the coast, (ii) a high prevalence of interspecific hybrids despite clear morphological species differences, and (iii) long-term, low level hybridization. These results are based on multiple lines of evidence from genetic diversity, phylogenetic trees and coalescent analysis. Higher intraspecific nucleotide diversity in Salish Sea populations despite lower haplotype diversity was most likely explained by interspecific introgression. The paraphyly of species in the gene trees and the spatial pattern of nonconforming haplotypes also supported introgressive hybridization within the Salish Sea. The strongest support for introgressive hybridization comes from the congruent results of the coalescent and structure analysis where each showed high introgression from *S*. *maliger* into *S*. *caurinus* and *S*. *auriculatus* in Salish Sea populations. We found little evidence for hybridization in coastal populations with only some introgression of *S*. *caurinus* into *S*. *maliger*, but Bayesian assignment tests in *Structure* showed that over 30% of individuals in The Salish Sea were of hybrid origin. No F1 hybrids were detected (Fig 5, S2 and S4 Tables, and GenBank Accession JX886053-JX886620)., suggesting long term, low-level gene flow within the Salish Sea.

These results add to the increasing body of evidence demonstrating the uniqueness of Salish Sea populations of marine species and the dynamic nature of evolutionary processes within this relatively enclosed area. Hybrids were discovered in the Salish Sea, but were very rare on the outer coast. Physical and oceanographic forces appear to prevent dispersal of hybrids out of the Salish Sea thus retaining introgressed alleles, especially in more isolated basins. All three species have been reported to be genetically different in the Salish Sea Basin compared to their respective coastal populations [28, 29, 31, 34], possibly in part because of hybridization. *Sebastes auriculatus* in the Salish Sea are much more physically isolated from their coastal counterpart populations than the other two species, because they are rarely found along the Pacific Ocean coast from Oregon to British Columbia [31, 35, 57]. Correspondingly, our phylogenetic analysis of mitochondrial DNA showed significant population differentiation for *S*. *auriculatus* in the Salish Sea compared with coastal *S*. *auriculatus* (Fig A in S1 File). Recruitment of juvenile rockfish from outside the main basin appears to be limited, due to low surface-water exchange from outside the main basin as estimated by drift card studies [33]. In addition, gene flow may be limited by selection against immigrants, as shown in replicate coastal and Sound populations of *S*. *caurinus* on Vancouver Island [58]. Limited intraspecific gene flow with the coast may allow introgressed alleles to accumulate in Salish Sea populations of *S*. *auriculatus* and *S*. *caurinus*; in contrast, *S*. *maliger* from the Salish Sea may actually be more connected to their coastal populations. In fact, the only two hybrids from the coastal populations were *S*. *maliger* backcrosses, collected near the entrance of the Salish Sea near the Strait of Juan de Fuca. Furthermore, genetic evidence for limited dispersal from the Salish Sea has been documented not only in rockfish [28, 34], but also in clams, *Protothaca staminea* and *Macoma balthica* [59], Pacific cod [60], and Pacific hake [61].

Within the Salish Sea, the proportion of hybrids increased with geographic isolation from the outer coast (Fig 6). Natural barriers to dispersal are likely found at two major shallow sills at Admiralty Inlet (between NPS and CPS) and the Tacoma Narrows (between CPS and SPS); also in addition, shallow sills separate Whidbey Basin and Hood Canal from the main basin [31, 35, 62]. Our results suggest that hybridization is predominantly occurring in these isolated bays in the Salish Sea, and that hybrids are retained within those environments. Alternatively, there may be a hybrid advantage in enclosed bays that is less pronounced in the larger basins of the Salish Sea. Such spatially varying dynamic processes may explain the maintenance of distinct morphological species despite the high prevalence of hybridized individuals. We are unable to completely rule out that the introgression in the Salish Sea is due solely to retention of hybrid events when the Salish Sea was formed.

Opportunities for hybridization in the Salish Sea compared with those on the outer coast are likely increased by limited habitat in the Puget Sound main basin, which has 20 times less reef habitat compared to the Strait of Juan de Fuca and the San Juan Islands north of Puget Sound Basin [37]. Most rockfish species, including the three species investigated here are closely associated with rocky reef habitats. The limited available rocky reef habitat in Puget Sound may increase spatial overlap between species and thus provide more opportunity for hybridization. Such habitat constraints may be reinforced by other environmental factors: compared to the coast, Puget Sound has naturally lower salinity, more variable temperatures, and sporadic anoxic conditions [31, 35]. The Puget Sound sub-basins experience anoxic conditions more often than the main basin because they can be strongly stratified by heavy, seasonal freshwater input [63]. Such oxygen depletion generally starts in deep waters and forces species into shallower habitat. Many rockfish species are segregated by their depth preference [30, 64]; for example, *S*. *maliger* is usually found at the deepest distribution while *S*. *auriculatus* occurs at more shallow depths [32, 35]. Periodic low oxygen levels, which occur frequently in Hood Canal [65] may force *S*. *maliger* into more shallow depths, which provides more opportunities for hybridization due to the increased contact with *S*. *caurinus* and *S*. *auriculatus*. Such forced migration may also explain patterns of asymmetric introgression if hybrids stay in shallow water while pure *S*. *maliger* retreat back to depth when oxygen levels return to normal. Habitat constraints and environmental conditions may thus provide opportunities for hybridization and may also explain the directional patterns of hybridization.

The highly asymmetrical patterns of introgression may also be caused by colonization history differences in abundance and mating behavior. Rockfishes presumably colonized the Salish Sea as the glaciers receded at the end of the Pleistocene. Habitat characteristics described above may have led to secondary contact between species that are otherwise separated by depth,such secondary contact is often seen as the primary factor leading to introgressive hybridization and the formation of hybrid zones [12]. Hubbs [66] postulated that stark differences in abundance between species (such as invader and resident species) may lead to hybridization and introgression from the more common to the rarer species. If the three species colonized the Salish Sea at different times, later colonizers may have been introgressed by already established species, as suggested in computer models and empirical data [67]. Indeed, *S*. *maliger*, the species with the largest population size estimates in the Salish Sea from coalescent analysis (Fig 2) appeared to have introgressed into the other two species. Recent (1999–2004) population size estimates from biological surveys [57] suggest about equal population sizes of *S*. *maliger* and *S*. *caurinus*, while *S*. *auriculatus* is absent from the North Sound and less abundant than the other two species in the South Sound. However, all three species experienced strong declines since the 1970s [57], and coalescence estimates tend to integrate over extended time periods [68]. *Sebastes maliger* have the longest generation times and lowest productivity of the three species [57], and so may have been more affected than *S*. *caurinus* and *S*. *auriculatus* by the recent declines, suggesting that *S*. *maliger* was indeed the most abundant species before the onset of commercial and recreational fisheries in the Salish Sea. Introgression was strongest into Salish Sea populations of *S*. *auriculatus*, which has the lowest abundance of the three species estimates both from coalescence (Fig 2) and from biological surveys [57].

Another explanation for the asymmetric introgression in these species may be mating behavior or differential fitness of hybrids. Little is known about the mating behaviors of rockfishes though there is the potential of mate choice given internal fertilization. The asymmetrical pattern of introgression from *S*. *maliger* into the other two species could be explained by hybrid mate selection, in which F1 hybrids preferentially mate with *S*. *caurinus* or *S*. *auriculatus* and rarely with *S*. *maliger*. On the other hand, asymmetrical introgression may also be due to reduced fitness in some hybrid crosses [69]. Biological factors as well as mating behaviors or hybrid fitness are also likely influencing the patterns of introgression in the Salish Sea. *Sebastes caurinus* and *S*. *maliger* are more closely related to each other than to *S*. *auriculatus* [30], and hybrids between closely related taxa may be more viable than those between distantly related species. Indeed, we found more introgression between *S*. *maliger* and *S*. *caurinus*; however, relatedness between these species does not entirely explain the asymmetry of introgression into *S*. *caurinus*.

The direction of introgression we detect from *S*. *maliger* into the other two species is in contrast to previous reports that allozyme alleles characteristic for the other two species were found in Salish Sea *S*. *maliger* [28]. This discrepancy to our results could be due to retention of some allozyme polymorphisms since speciation or selection at allozyme loci. Similar to Seeb (1986), however, we found extensive introgression of *S maliger* mitochondrial DNA into the other two species. Another study based on microsatellites and assignment tests suggested that *S*. *caurinus* was not impacted by hybridization [29]. However, microsatellites can be problematic in hybridization studies because of extensive homoplasy [70], and our *Structure* plot (Fig 5) suggested that most loci in hybrids conform to the morphological species identification. Both these studies also had more limited sampling within the Salish Sea. Nevertheless, a consistent analysis of these samples may provide interesting insights into the temporal dynamics of hybridization over three decades. Similar studies have shown a surprising stability of a hybrid swarm in Australian estuarine bream species, suggesting rare hybridization events that lead to the formation of temporally stable hybrid swarms [71].

The more information on timing of hybrid events the better our predictions are for recent anthropogenic changes in the Salish Sea. We found zero F1 hybrids and hybridization was detected at only one or two loci (S2 Table), confirming, first, that initial hybrids and their backcrosses were fertile, and second, that introgressive hybridization is the result of many generations of backcrosses. These patterns suggest that hybridization was not the result of recent anthropogenic changes in the Salish Sea. The Puget Sound shoreline alone is home to over 1.1 million people with over half of the area developed as urban or agriculture landscape [62]. Agricultural and urban run-off may have exacerbated the natural tendency for anoxic conditions, similar to Cheasapeake Bay and the Gulf of Mexico, thus potentially increasing habitat overlap and opportunity for hybridization between the three species. These anthropogenic effects were most extreme in the past 3–5 generations (mean generation time for the three species 8–20 years [35], and if they were the primary cause of hybridization, detection of some F1 hybrids would have been expected. Instead, our results suggesting long term, low level hybridization correspond to findings that anoxic conditions in Hood Canal are a natural phenomenon and may have already been prevalent in the 17^th^ and 18^th^ centuries [72].

Even though a high frequency of hybrids in the Salish Sea *S*. *caurinus* and *S*. *auriculatus* was detected, the true frequency may be even higher given the limited number of markers employed and their ability to resolve all three species. If introgressive hybridization has been ongoing for generations in the Salish Sea, then more markers would increase the detection power for later generation hybrids [73]. We would further expect that increased detection would reinforce the geographic patterns seen within the Salish Sea. The uneven sampling between populations from the outer coast and the Salish Sea of conspecifics were considered; however, the coalescent analyses showed very similar population size estimate between populations. The genetic information was sufficient to evaluate introgression in our study with 92 SNPs. Although there were a limited number of independent loci we improved the power by using linked loci [52].

Although we found extensive introgression in the Salish Sea, this hybrid zone does not appear to be a hybrid swarm because retention of parental morphological types is evident. Rather than seeing evidence of two or three gene pools fusing in the Salish Sea *Sebastes*, most hybrids were morphologically and genetically similar to one of the three species, although some fish with intermediate morphology are found in the Salish Sea *Sebastes*, as seen here and in other studies [28]. This suggest that there are prezygotic (mating behavior, gamete recognition) or postzygotic (reduced hybrid fitness) mechanisms that prevent random interbreeding and the formation of a hybrid swarm. Such mechanisms may lead to reinforcement that aids speciation during the early stages and may lead to hybrid speciation [74]. Hybridization is a relatively common phenomenon in *Sebastes* [75–80], and the rapid speciation in the group may be linked to such hybridization events. Furthermore, introgression can serve as an abundant and faster source of genetic variation than mutation [81] and so can aid adaptation even if initial hybridization is maladaptive [74].

Future work on Salish Sea rockfish hybrids needs to include next generation sequencing to capture the entire genome, both to detect further hybrid backcrossed individuals and to explore specific genetic regions influenced by hybridization in these species. Interspecific gene flow might be temporarily or spatially restricted or it may occur in some parts of the genome but not in others [82–84]. Genetic loci with restricted gene flow in hybridizing species can provide insights on reproductive barriers in sympatric species [85]. The hybrid zone in the Salish Sea is an ideal location to investigate localized interspecific interactions, yet the species dynamics need to be explored in other areas of sympatry. Further research should include expanding the geographic scope to include additional regions with post Pleistocene glacial influence, such as Queen Charlotte Sound in British Columbia. Also further information about the effects of hybridization on morphology need to be accessed using known F1 and F2 hybrids. Understanding what and how morphological traits are influenced by hybridization will be critical to understand the biological response to hybridization in the wild.

## Supporting information

S1 File Details of phylogenetic results. (PDF)

S1 Table Detailed table of tissue samples. (XLSX)

S2 Table Individual samples with a discordant haplotype. (PDF)

S3 Table Population mutation rate (per gene per generation) used in coalescent analysis. (XLSX)

S4 Table The *structure* ancestry coefficient output (Q). (XLSX)

S5 Table Probability of equal hybrid proportion between Salish Sea and the coast. (XLSX)

S6 Table Probability of equal hybrid proportions across all regions. (XLSX)
