# Supplementary material for: Introgression among three rockfish species (Sebastes spp.) in the Salish Sea, northeast Pacific Ocean
Source: PLoS One. 2018 Mar 22;13(3):e0194068. doi: 10.1371/journal.pone.0194068 (PMC5864001; doi:10.1371/journal.pone.0194068)
Supplement: S1 File — (PDF) [file pone.0194068.s001.pdf]

Details of Phylogenetic Results

All morphological species were paraphyletic at all loci, though they tended to be monophyletic on the coast and paraphyletic in the Salish Sea. Monophyletic clades with moderate bootstrap support (>65%) containing all coastal individuals were found for *S. auriculatus* and *S. caurinus* at *Cytb* (Figure A) and *S. auriculatus* at *Ets* (Figure B) and *S7* (Figure C). Monophyletic clades, though with low bootstrap support, were also found for coastal *S. auriculatus* at *Mep* (clade G, Figure D) and coastal *S. maliger* at *Cytb* (clade B, Figure A). In contrast, Salish Sea populations had more shared haplotypes and discordant haplotypes. At *Ets*, *Mep*, and *Mdh*, haplotypes shared among the three focal species were also shared with an ingroup control or the outgroup species (Figures B, D, and E).

The *Cytb* tree had 30 unique haplotypes with strong bootstrap support for two distinct clades (A and C, Figure A). The *S. Caurinus,* clade C had 92% bootstrap support and grouped most morphological *S. caurinus*. The nine discordant haplotypes were Salish Sea *S. caurinus* and were shared with a common coastal *S. maliger* haplotype (haplotype18, clade B, Figure A, S3 Table). The *S. auriculatus,* clade A had 86% bootstrap support and included all but two haplotypes from the morphological *S. auriculatus*. Those discordant haplotypes (haplotype 20 and 23) were from Salish Sea *S. auriculatus* and clustered with common *S. maliger* haplotypes (clade B, Figure A, S3 Table), though haplotype 23 was not shared between the two species. Interestingly, clade A2 grouped all Salish Sea *S. auriculatus* with 75% bootstrap support (Figure A), with the exception of the two non-conforming haplotypes mentioned above. Clade B grouped the majority of *S. maliger* morphological species (Figure A) except for a few discordant haplotypes from Salish Sea *S. maliger* that were shared with *S. auriculatus* (clade A) and *S. caurinus* (clade C) (haplotypes 22 and 12, Figure A, S3 Table).

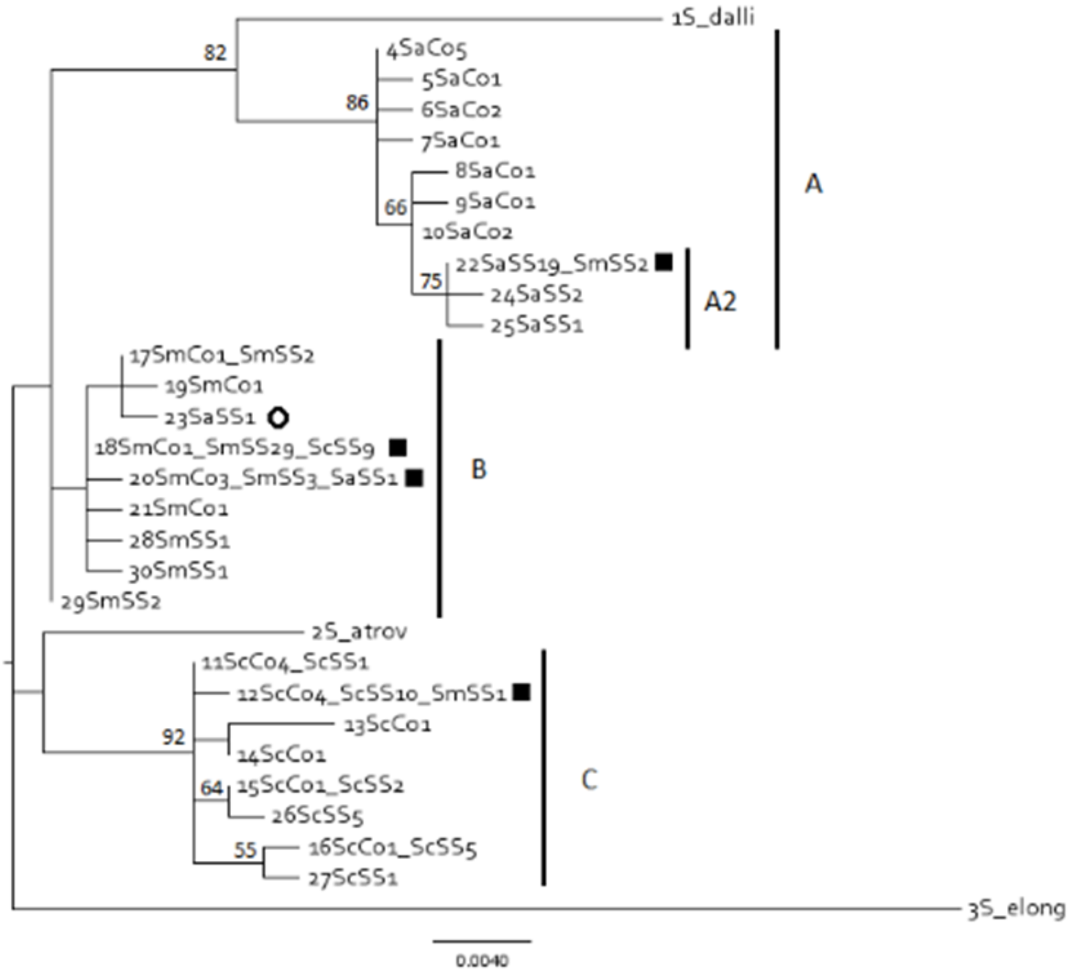

Figure A. *Cytb* (717 nucleotides and HKY+G mutation model). Maximum likelihood phylogenetic tree with >50% support indicated beside branch. Node label coded with the following information: unique haplotype identifier, species, population, haplotype count. Species are coded by two letters: species (Sa–*S. auriculatus,* Sc–*S. caurinus,* Sm–*S. maliger*). The next two letters are the population code (Co – Coastal, SS – Salish Sea). The discordant haplotypes that were shared are marked with a solid square and clades are labeled by the letters A, A2, B, and C. Open circle represents discordant haplotype that was not shared. The tree is rooted with outgroup *Sebastes elongatus*.

Trees from nuclear loci were generally less resolved among coastal morphological species, complicating the distinction of hybridization and ancestral polymorphism. The *Ets* tree had 15 haplotypes with 68% support for clade D that grouped all morphological *S. auriculatus* (Figure B). Morphological *S. maliger* and *S. caurinus* shared one haplotype with each other (haplotype 3, Figure B) and one haplotype both with each other and the ingroup control *S. atrovirens* (haplotype 8, Figure B). Although morphological *S. maliger* and *S. caurinus* were not completely resolved into monophyletic clades, haplotype 3 was mostly *S. caurinus* and haplotype 8 was mostly *S. maliger*.

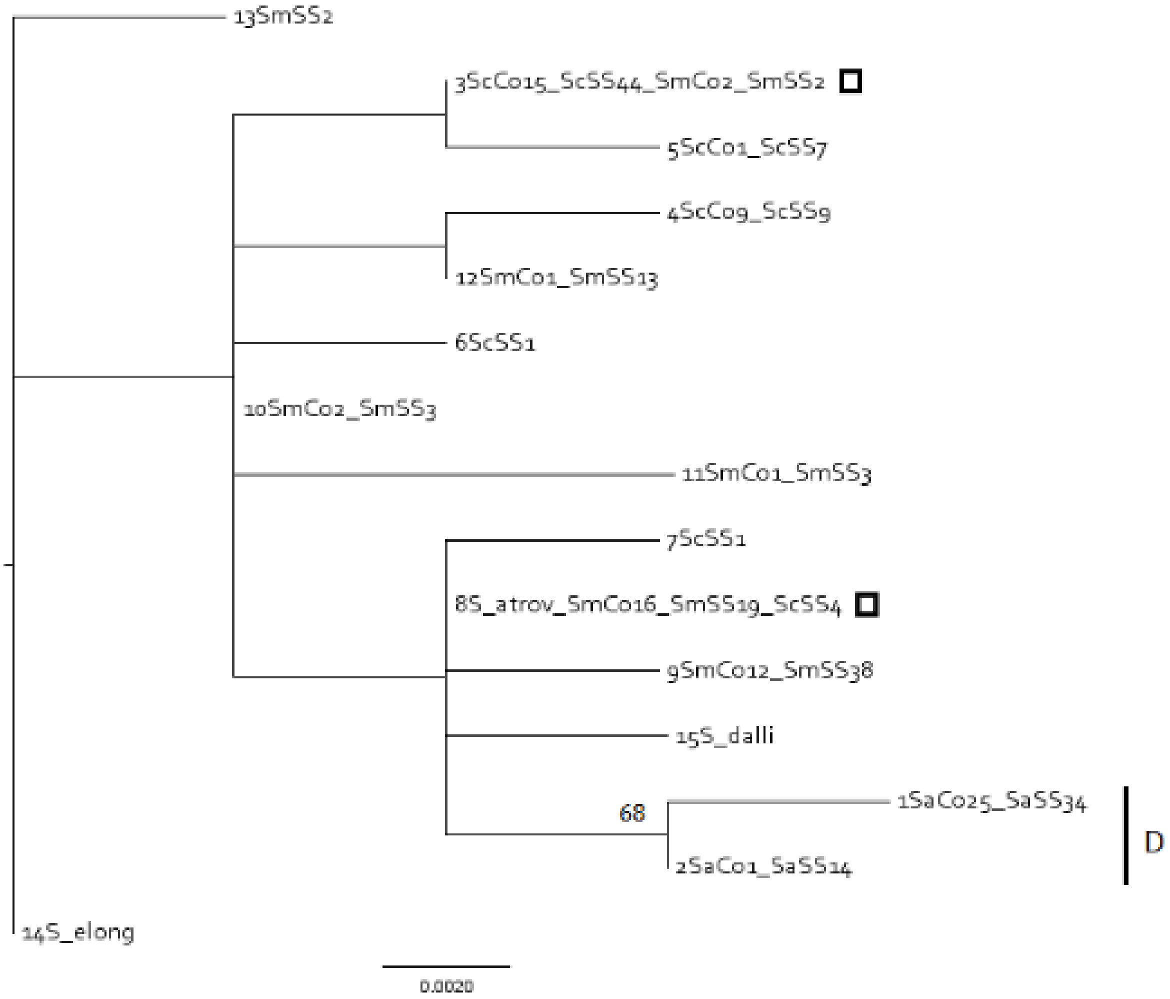

Figure B. *Ets* (293 nucleotides and K80+I mutation model). Maximum likelihood phylogenetic tree with >50% support indicated beside branch. Node label coded with the following information: unique haplotype identifier, species, population, haplotype count. Species are coded by two letters: species (Sa – *S. auriculatus,* Sc – *S. caurinus,* Sm – *S. maliger*). The next two letters are the population code (Co – Coastal, SS – Salish Sea). The shared haplotypes are marked with an open square and the distinct clade is labeled with D. The tree is rooted with outgroup *Sebastes elongatus*.

The *S7* tree had 20 haplotypes with 97% support for clade F that grouped all morphological *S. auriculatus* haplotypes except two and the ingroup control *S. dallii* (Figure C). The two discordant haplotypes were Salish Sea *S. auriculatus* that were shared with common coastal *S. maliger* haplotypes (4 and 6, Figure C). Clade E grouped most morphological *S. caurinus* haplotypes except for six discordant haplotypes that grouped with common coastal *S. maliger* haplotypes (4 and 5, Figure C). Although haplotypes from morphological *S. maliger* did not form a distinct clade at *S7*, they were not grouped with the other morphological species in clade F or E, with the exception of a single haplotype from a coastal *S. maliger* found in clade E (Figure C).

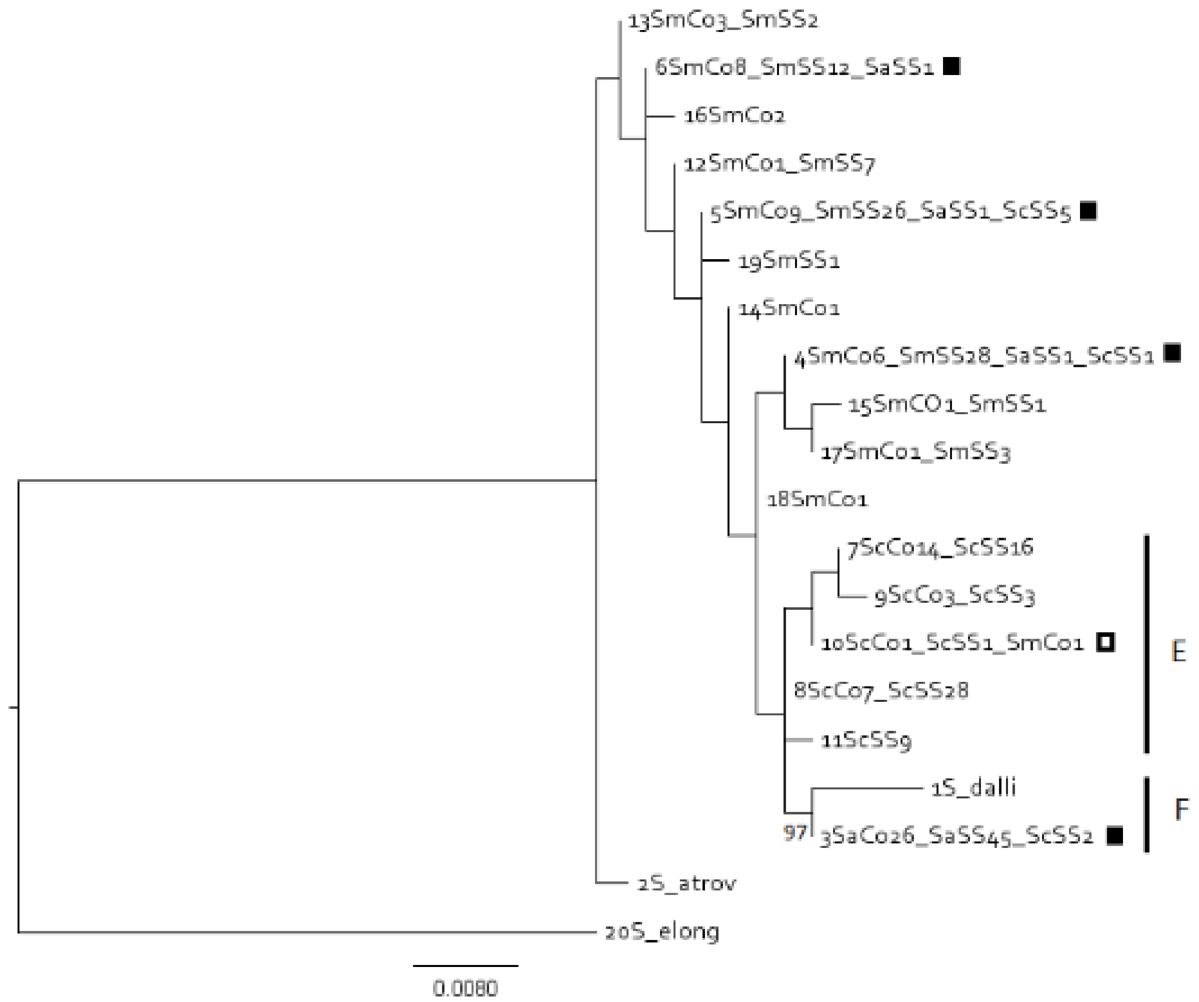

Figure C. *S7* (560 nucleotides and F81+I+G mutation model). Maximum likelihood phylogenetic tree with >50% support indicated beside branch. Node label coded with the following information: unique haplotype identifier, species, population, haplotype count. Species are coded by two letters: species (Sa – *S. auriculatus*, Sc – *S. caurinus,* Sm – *S. maliger*). The next two letters are the population code (Co – Coastal, SS – Salish Sea). The discordant haplotypes that were shared are marked with a solid square and distinct clades are labeled E and F. The open square marks a shared haplotype. The tree is rooted with outgroup *Sebastes elongatus*

The *Mep* tree had 32 haplotypes (Figure D) with clade G that grouped most haplotypes from morphological *S. auriculatus* and the ingroup control, *S. dallii*. Three shared haplotypes were from Salish Sea *S. auriculatus* and did not cluster with the rest of the morphological species in clade G (5, 9, 28, Figure D), though haplotype 28 was a unique haplotype.

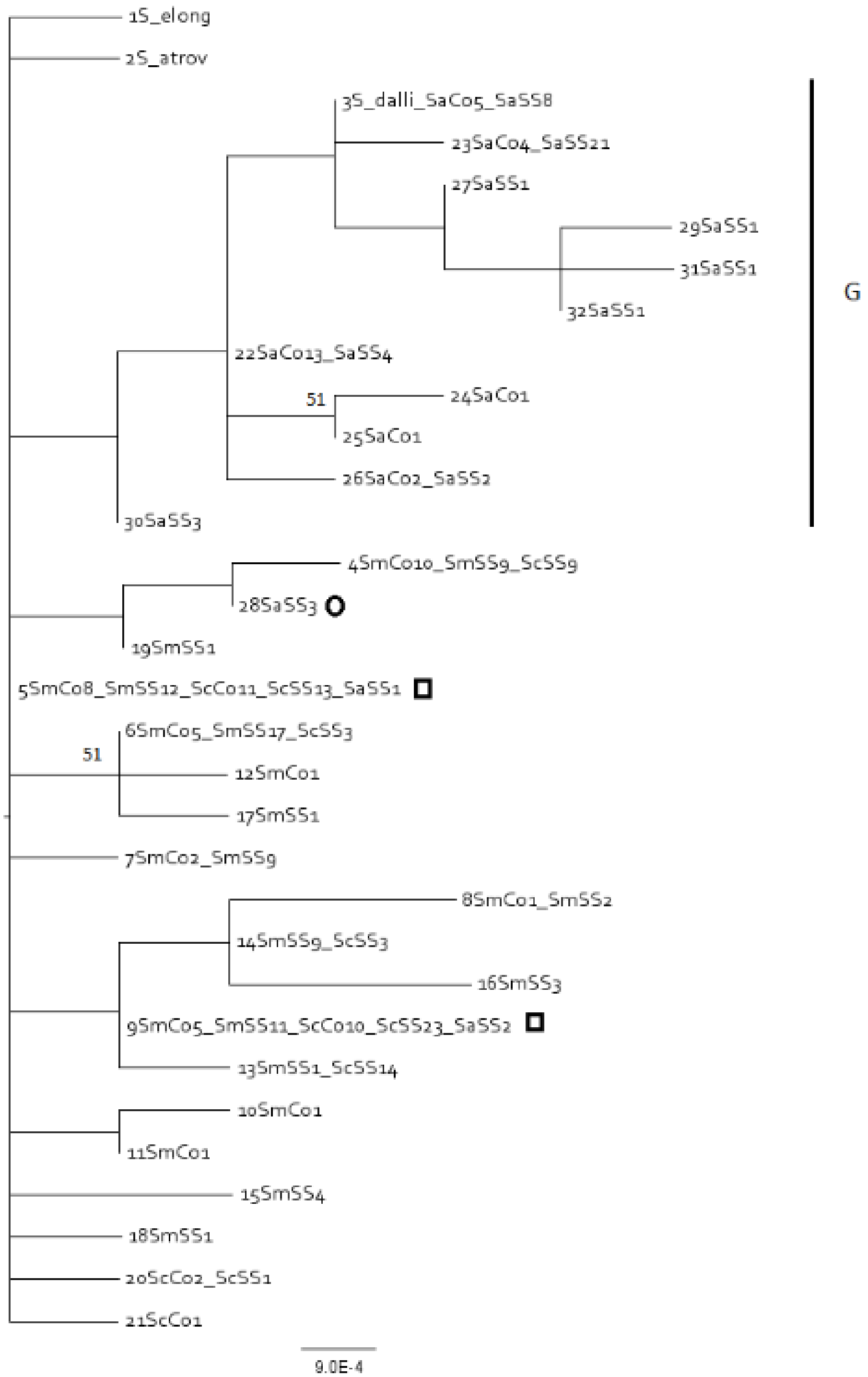

Figure D. *Mep* (785 nucleotides and HKY+I+G mutation model). Maximum likelihood phylogenetic tree with >50% support indicated beside branch. Node label coded with the following information: unique haplotype identifier, species, population, haplotype count. Species are coded by two letters: species (Sa – *S. auriculatus*, Sc – *S. caurinus,* Sm – *S. maliger*). The next two letters are the population code (Co – Coastal, SS –Salish Sea. The discordant species haplotypes are marked with a solid square and the distinct clade is labeled G. The open circle represents a discordant haplotype that was not shared. The open square marks a shared haplotype. The tree is rooted with outgroup *Sebastes elongatus*.

The *Mdh* tree had 8 haplotypes and appeared to be polyphyletic for all morphological species; however, there is a single shared haplotype that represented almost all the morphological *S. caurinus* (2, Figure E). The *S. caurinus* shared this haplotype with two coastal *S. maliger* morphological species, one Salish Sea *S. auriculatus*, and the outgroup *S. elongatus*. The nine haplotypes from Salish Sea *S. caurinus*, which did not share haplotype 2, were shared among two common haplotypes (1 and 3) found in the other morphological species plus both ingroup controls (Figure E).

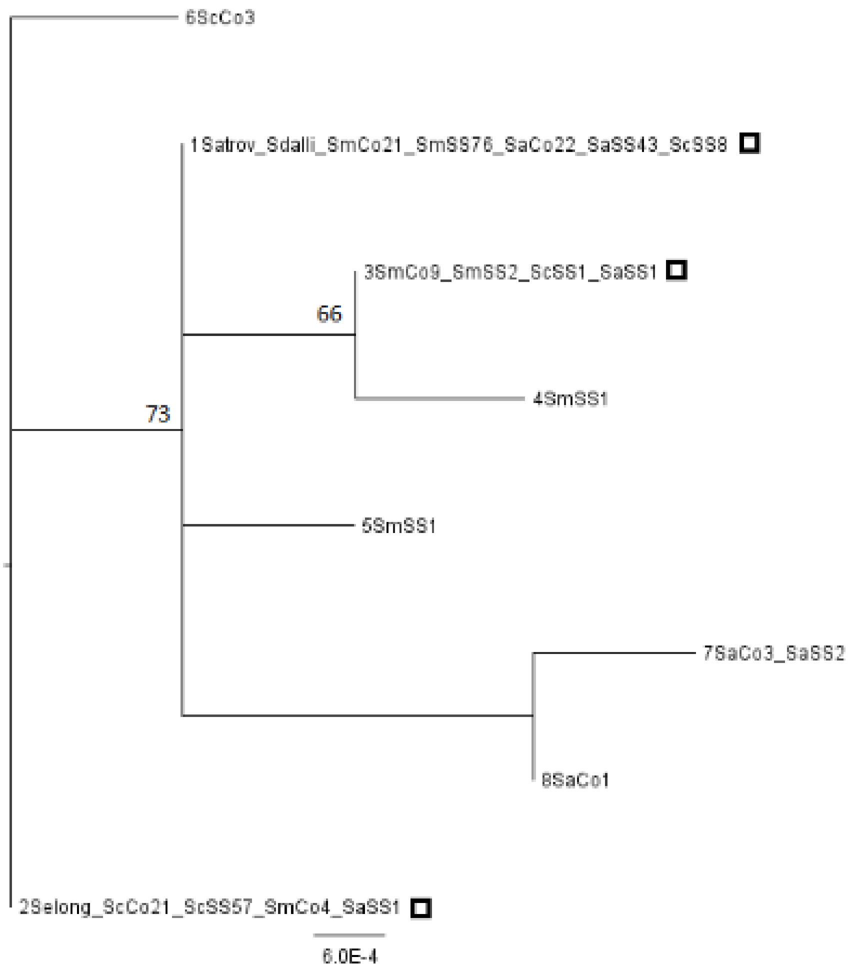

Figure E. *Mdh* (730 nucleotides and HKY mutation model). Maximum likelihood phylogenetic tree with >50% support indicated beside branch. Node label coded with the following information: unique haplotype identifier, species, population, haplotype count. Species are coded by two letters: species (Sa – *S. auriculatus*, Sc – *S. caurinus*, Sm – *S. maliger*). The next two letters are the population code (Co – Coastal, SS - Salish Sea). Shared haplotypes are marked with an open square. The tree is rooted with outgroup *Sebastes elongatus*.
