# Supplementary material for: Introgression among three rockfish species (Sebastes spp.) in the Salish Sea, northeast Pacific Ocean
Source: PLoS One. 2018 Mar 22;13(3):e0194068. doi: 10.1371/journal.pone.0194068 (PMC5864001; doi:10.1371/journal.pone.0194068)
Supplement: S2 Table — (PDF) [file pone.0194068.s003.pdf]

S3 Table. Individual samples with a discordant haplotype (gray highlight) or individuals whose haplotypes are 'shared' haplotypes with another morphological species. The columns for each locus see Cytb (S2 File,Figure A); Ets (S2 File,Figure B); S7 in (S2 File,Figure C); Mep (S2 File,Figure D); Mdh (S2 File,Figure E). The Fish Identifier column abbreviates species and population as follows: Sa – *S. auriculatus*, Sc – *S. caurinus*, Sm – *S. maliger*, Co – coast, and SS –Salish Sea. The number in each column is the haplotype identifier for that locus and that individual. The 'u' designates where the haplotype is unique otherwise they were all 'shared'. The nuclear loci show both haplotypes with a '/' separator if the second haplotype conforms to morphological species then '/ – .'

Fish Identifier	Region	CYTB	ETS	S7	MEP	MDH
SaSS008	SPS	23 u [M]				
SaSS011	CPS				23 u/ –	
SaSS012	CPS			5/ – [M]		
SaSS023	CPS				5/9	2/2
SaSS025	HC			5/6 [M]		
SaSS006	SPS				23 u/ –	
SaSS004	SPS	20 [M]			9/ –	
SaSS017	SPS				23 u/ –	
ScSS051	CPS			4/ – [M]		
ScSS053	CPS			5/ – [M]		
ScSS054	CPS					1/ –
ScSS055	CPS		8/8	3/ – [A]		
ScSS056	CPS	18 [M]		3/ – [A]		
ScSS063	HC		8/8			
ScSS065	HC	18 [M]				
ScSS069	HC	18 [M]				
ScSS071	HC	18 [M]		5/ –		
ScSS073	HC			5/ –		1/ –
ScSS074	HC					1/ –
**ScSS045**	SPS	18 [M]		5/ –		1/ –
ScSS046	SPS			5/ –		
ScSS048	SPS	18 [M]				1/ –
ScSS087	NPS					3/ –
ScSS002	WB	18 [M]				
ScSS033	WB	18 [M]				
ScSS035	WB	18 [M]				1/ –
ScSS039	WB					1/ –
ScSS040	WB					1/ –
SmSS181	SPS	12 [C]				
SmSS161	WB	22 [A]				
SmSS164	WB	22 [A]				
SmSS173	WB		3/3			
SmCo019	Coast		3/3			2/2
SmCo022	Coast			10/ –		2/2

[M] haplotype is most common in *S. maliger* morphological species, [A] haplotype is most common in *S. auriculatus* morphological species, [C] haplotype is most common in *S. caurinus* morphological specie
